# Prevalence, molecular characterization of integrons and its associated gene cassettes in *Klebsiella pneumoniae* and *K. oxytoca* recovered from diverse environmental matrices

**DOI:** 10.1038/s41598-023-41591-7

**Published:** 2023-09-01

**Authors:** Folake Temitope Fadare, Taiwo Olawole Fadare, Anthony Ifeanyi Okoh

**Affiliations:** 1 SAMRC Microbial Water Quality Monitoring Centre, University of Fort Hare, Alice, South Africa; 2 Applied and Environmental Microbiology Research Group (AEMREG), Department of Biochemistry and Microbiology, University of Fort Hare, Alice, South Africa; 3 Department of Microbiology, Obafemi Awolowo University, Ile-Ife, Osun State Nigeria

**Keywords:** Microbiology, Environmental sciences

## Abstract

The high prevalence of infections arising from *Klebsiella* species is related to their ability to acquire and disseminate exogenous genes associated with mobile genetic elements such as integrons. We assessed the prevalence, diversity, and associated gene cassettes (GCs) of integrons in *Klebsiella* species. The isolates recovered from wastewater and hospital effluents, rivers, and animal droppings were identified using the conventional Polymerase Chain Reaction (PCR) with primers targeting the *gryA*, *pehX*, and *16S–23S* genes. The antimicrobial resistance profile and the Extended-Spectrum and Metallo β-lactamases production were carried out using standard microbiological techniques. PCR, DNA sequencing analyses, and Restriction Fragment Length Polymorphism were used to characterize the integrons and their associated GCs. Furthermore, the genotypic relationships between the different isolated *K. pneumoniae* were determined using Enterobacterial Repetitive Intergenic Consensus (ERIC)-PCR. About 98% (51/52) of the confirmed isolates harboured an integrase gene, with 80% *intI1*, while the remaining 20% concurrently harboured *intI1* and *intI2*, with no *intI3* observed. About 78% (40/51) of the bacterial strains were positive for a promoter, the *P2R2*, investigated, while 80% (41/51) harboured at least one of the *qacEΔ1* and *sul1*. Three different GCs arrangements identified were *aac(6′)-Ib, aadA1*-*dfrA1*, and *dfrA1*-*sat2*. At a similarity index of 60%, the ERIC-PCR fingerprints generated were categorized into nine clusters. Our study is the first to reveal the features of integrons in *Klebsiella* spp. recovered from environmental sources in the Eastern Cape Province, South Africa. We conclude that the organisms' sources are repositories of integrons harbouring various gene cassettes, which can be readily mobilized to other microorganisms in similar or varied niches.

## Introduction

The *Klebsiella* genus is a Gram-negative bacteria becoming implicated as opportunistic pathogens linked to severe hospital-acquired illnesses, including urinary tract infections, septicaemia, and pneumoniae^1, 2^. The most common species in this genus are *K. oxytoca* and *K. pneumoniae*, accounting for approximately 8% of all hospital-acquired infections in Europe and the United States^3^. Antibiotics are generally given to treat the illnesses they cause. Unfortunately, over the decades, the increased use of antibiotics in veterinary, agricultural, and clinical sceneries has aggravated the emergence and widespread of multidrug-resistant (MDR) pathogens, even in environmental sources^4^. This scenario has reduced treatment options with current antibiotics, especially when MDR bacteria harbour genes conferring resistance to extended-spectrum β-lactamases (ESBLs) and carbapenemases. Due to the pressure exerted by the widespread use of antibiotics, MDR *K. pneumoniae* has often been implicated in hospital endemics making treating hospital-associated infections difficult, especially strains resistant to carbapenems^5, 6^. In the past few years, the environment has been seen as a critical factor in the spread of these organisms. An important probable reason is the possible selection pressure caused by antibiotic residues in wastewater^7^. *K. pneumoniae* is one of the microorganisms that can readily acquire the genes that produce hydrolyzing enzymes, which can be harboured on various mobile genetic elements, such as integrons, further accelerating their dissemination process^2^.

Integrons are mobile genetic elements found on pathogenicity islands, transposons, and plasmids, easing their distribution among various bacteria^8, 9^. They are considered efficient gene expression systems that naturally capture, integrate gene cassettes (GCs) and also provide a promoter which has the propensity for the expression of the captured antimicrobial resistance genes (ARGs) on the GCs^10–13^. All integrons have a functional platform on the 5′-Conserved Segment (CS) that harbours the integron-integrase, *intI* gene, the integron-associated recombination site, *attI*, and a highly constitutive promoter, P_c_^10, 14^. Integrons are categorized into classes according to the sequences of the *intI* gene^15, 16^. The classes often detected within the *Enterobacteriaceae* family include class 1, class 2, and class 3 integrons^17^. The most prevalent class of integrons is class 1 with *intI1*, frequently reported in Gram-negative bacteria^2, 18^. Typical class 1 integrons have a second CS known as the 3′ CS with a typical sequence length of 2384 bp and encodes four open reading frames (ORFs)^17, 19^. The first ORF harbours the *qacE* that encodes resistance to quaternary ammonium compounds, followed by the ORF harbouring *sul1*, conferring resistance to sulphonamides. Other ORFs include the *orf5*, a gene whose exact function is unknown but with some similarity to puromycin acetyltransferase and the *orf6*, with a yet-to-be-discovered biological activity^13, 20, 21^. Non-typical class 1 integron lacks the 3′ CS region as well as the ORFs, *qacE*, *sul1, orf5* and *orf6,* present in a typical class 1 integron^8, 22, 23^. Class 2 integrons harbour the *intI2*, whose sequences share a 46% similarity with *intI1*. The distinctive feature of *intI2* is that it is shortened due to a stop codon at the 179 bp position responsible for the low variety of GCs and, consequently, its less involvement with resistance genes^16, 17, 24, 25^. Although, a few functional class 2 integrons have been reported^16, 26, 27^. In Gram-negative bacteria, class 2 integrons are less prevalent when compared to class 1 integrons, while the class 3 integrons with *intI3* are the least reported among the *Enterobacterales*^2, 18, 28^.

The integron-encoded integrase can recombine discrete units of circularized DNA called gene cassettes^29^. Gene cassettes are variable sequences that can occur as free, circular, nonreplicating DNA molecules and are usually linear when integrated into integrons^15^. They are composed of an ORF and a recombination site, *attC*. The *intI* catalyzes the *attI* and the *attC* site recombination^15, 29, 30^. The presence of GCs has been known to confer resistance to various antibiotic classes. These include aminoglycosides, erythromycin, antiseptics of the quaternary ammonium compounds, β-lactams, chloramphenicol, fosfomycin, trimethoprim, lincomycin, quinolones, rifampicin, and streptothricin^17, 25, 29, 30^. Integrons harbouring several cassette arrays have been described in continents such as North and South America, Europe, and Asia^2, 17, 28^. Although the main focus of detecting integrons and their GCs was within the clinical environment, they have also been identified in several bacterial species from various environmental sources^14, 22, 29^. In Africa, some studies have reported the detection of GCs within the environment^31, 32^ however, no studies exist on integron characterization in *Klebsiella* spp. from environmental sources in the Eastern Cape Province (ECP) in South Africa. This study reports the prevalence and characteristics of integrons and associated GCs in *Klebsiella* species from various environmental sources in the ECP in South Africa.

## Materials and methods

### Bacterial strains

Fifty-two *Klebsiella* species isolates recovered from various environmental matrices in Amathole and Chris Hani District Municipalities in the ECP were used in this study. Twenty-nine of the isolates used in this current study were part of our earlier studies. The description of the study sites, sample collection and processing, and isolation were carried out as reported in our previous studies^33–35^. The isolates in this study include, 33 *Klebsiella pneumoniae*recovered from freshwater sources (n = 7), hospital wastewater (HWW) (n = 9), and wastewater treatment plant effluents (WWTP) (n = 7), while the remaining were isolated from animal droppings (FD). In addition, 19 K*. oxytoca* were recovered from HWW (n = 11), WWTP (n = 2), and FD (n = 6). The bacterial isolates were recovered between October and November 2017. The bacterial isolates were stored at – 80 °C in 20% glycerol stock as part of the Applied and Environmental Research Group (AEMREG) culture collection. Ethical clearance was obtained from the University of Fort Hare Research Ethics Committee with reference number REC-270710-028-RA Level 01 to access these bacterial cultures.

### Resuscitation and DNA extraction of bacterial cultures

The retrieved glycerol stocks were resuscitated in Brain Heart Infusion (BHI) broth (Merck, South Africa) and incubated at 37 °C for 18 h. A loopful was streaked on nutrient agar (Oxoid, UK) and purified twice. For subsequent analysis, single pure colonies were transferred to 2 ml BHI broth and incubated overnight at 37 °C. The boiling method^36^ was used to extract the genomic DNA with slight modifications, as previously reported elsewhere^35^. The DNA-containing supernatant was then transferred to sterile microcentrifuge tubes (Eppendorf, Germany) and stored at – 20 °C for future assays.

### PCR-based confirmation of *Klebsiella* spp.

The molecular confirmation of the *Klebsiella* genus was by the conventional Polymerase Chain Reaction (PCR) assay using primer sets that target the *gryA* gene. All genus-confirmed *Klebsiella* isolates were speciated into *K. pneumoniae and K. oxytoca* using primers targeting the 16S–23S ITS and *pehX* genes, respectively. *K. oxytoca* NCTC 11686 was included as a positive control (Microbiologics, Medimark, France), while *E. coli* ATCC 8739 was used as the negative control following conditions described in Table 1.

**Table 1:** The list of primers and thermocycling conditions used for PCR amplification.

Primer	Primer Sequence (5′ → 3′)	Amplicon size (bp)	Thermocycling conditions	Reference
*gryA-*F *gryA-*R	CGCGTACTATACGCCATGAAGTA ACCGTTGATCACTTCGGTCAGG	441	94 °C for 5 min; 35 [94 °C, 30 s; 55 °C, 45 s; 72 °C, 45 s]; 72 °C for 7 min	^37^
*16S-23S ITS-*F *16S-23S ITS -*R	ATT TGA AGA GGT TGC AAA CGA T TTC ACT CTG AAG TTT TCT TGT GTT C	130	94 °C for 5 min; 30 [94 °C, 30 s; 55 °C, 30 s; 72 °C, 40 s]; 72 °C for 10 min	^38^
*pehX-*F *pehX-*R	GATACGGAGTATGCCTTTACGGTG TAGCCTTTATCAAGCGGATACTGG	343	94 °C for 5 min; 30 [94 °C, 30 s; 55 °C, 30 s; 72 °C, 40 s]; 72 °C for 10 min	^39^
*INTI1*-F *INTI1*-R	CAG TGG ACA TAA GCC TGT TC CCC GAG GCA TAG ACT GTA	164	94 °C for 5 min; 35 [94 °C, 60 s; 55 °C, 60 s, 72 °C, 30 s] 72 °C, 10 min	^40^
*INTI2*-F *INTI2*-R	TTATTGCTGGGATTAGGC ACGGCTACCCTCTGTTATC	232	94 °C for 5 min; 32 [94 °C, 60 s; 59 °C, 60 s; 72 °C, 2 min]; 72 °C for 10 min	^41^
*INTI3*-F *INTI3*-R	AGTGGGTGGCGAATGAGTG TGTTCTTGTATCGGCAGGTG	600	94 °C for 5 min; 32 [94 °C, 60 s; 48 °C, 60 s; 72 °C, 2 min]; 72 °C for 10 min	^41^
INTF P2R2	AGTGGGTGGCGAATGAGTG TGTTCTTGTATCGGCAGGTG	540	94 °C for 5 min; 35 [94 °C, 60 s; 55 °C, 60 s; 72 °C, 30 s]; 72 °C for 10 min	^42^
*qacEΔ1*-F qacEΔ1-R	ATC GCA ATA GTT GGC GAA GT CAA GCT TTT GCC CAT GAA GC	225	94 for 9 min; 30 [94 °C, 30 s; 55 °C, 30 s; 72 °C, 60 s]; 72 °C for 10 min	^20^
*Sul1*-F *Sul1*-R	ATGGTGACGGTGTTCGGCATCTGA CTAGGCATGATCTAACCCTCGGTCT	840	94 for 9 min; 30 [94 °C, 30 s; 55 °C, 30 s; 72 °C, 1 min]; 72 for 10 min	^43^
5′CS 3′CS	GGCATCCAAGCAGCAAG AAGCAGACTTGACCTGA	Variable	94 °C, 5 min; 35 [94 °C, 1 min; 45 °C, 1 min; 72 °C, 2 min]; 72 °C for 10 min	^44^
hep 74 hep51	CGGGATCCCGGACGGCATGCACGATTTGTA GATGCCATCGCAAGTACGAG	Variable	94 °C, 5 min; 33 [94 °C, 1 min; 59 °C, 45 s; 72 °C, 5 min]; 72 °C, 5 min	^11^

Each PCR mixture of a total of 25 µl contained PCR master mix (12.5 µl) (Thermo scientific (EU), Lithuania), forward and reverse primers (1 µl) (Inqaba Biotechnical Industries, South Africa). Furthermore, 5.5 µl nuclease-free water and a DNA template (5 µl) were added. The DNA amplification was done using a BioRad thermal cycler (LASEC, South Africa). A 1.5% (w/v) agarose gel stained with ethidium bromide (5 µl) was used to resolve 5 µl of the amplicons. The gel was run using Mupid-One (Eurogentec, Belgium) electrophoresis set-up at 100 V for 1 h in 0.5 × TBE buffer with a 100-base-pair molecular marker (Biolabs, New England). The results were viewed using UV transillumination (ALLIANCE 4.7, UVtec, London, UK).

### Antibiotic susceptibility, metallo and extended-spectrum β-lactamase production

The susceptibility profile was determined using the disk diffusion technique (DDT), approved by the Clinical and Laboratory Standard Institutes (CLSI). Isolates were assessed for ESBL production using the double-disk synergy test (DDST). It was done using ceftazidime-10 µg, cefoxitin-30 µg, and cefotaxime-30 µg in combination with amoxicillin/clavulanic acid-20 µg/10 μg. The isolates were also exposed to a panel of the following nine antimicrobial agents trimethoprim-sulfamethoxazole-1.25/23.75 μg, ampicillin-10 μg, colistin sulphate-10 μg, imipenem-10 g, chloramphenicol-30 μg, gentamicin-10 μg, ciprofloxacin-5 μg, nalidixic acid-30 µg, and tetracycline-30 μg. The antibiotics were purchased from Mast Diagnostics, South Africa.

The inoculum of *Klebsiella* spp. was suspended in a sterile saline solution with the turbidity adjusted to the 0.5 McFarland standard and evenly spread on Mueller–Hinton agar plates (Oxoid, UK). A disc dispenser (Mast Diagnostics, South Africa) was used to place the antibiotics. The plates were incubated at 37 °C for 18 h. After that, the inhibition zones' width was measured to the nearest millimetre and compared to the CLSI-established breakpoints to categorize the isolates as resistant, intermediate, or susceptible. Isolates resistant to more than two antimicrobial classes were considered MDR^45^.

The ethylenediaminetetraacetic acid (EDTA) test was used to determine the production of metallo β-lactamase (MBL) according to CLSI standards. A 0.5 McFarland adjusted test isolate was exposed to two 10 µg imipenem discs. Then, 10 µl of 0.5 M EDTA was added to only one disc to obtain a concentration of 750 µg, and an increase in inhibitory zone width of 5 mm in the disc potentiated with EDTA after 18 h of incubation at 37 °C was recorded as positive for MBL generation^45^.

### Molecular detection and characterization of integrons

The confirmed bacterial strains were screened with conventional PCR assays as described above. The presence of *intI1*, *intI2*, and *intI3* located on the 5′-CS was assayed for the classification of the integrons. In addition, *P2R2* on the 5′-CS of a typical integron was also screened to detect the presence of a promoter. Furthermore, the *intI1*-positive strains were assessed for genes (*qacEΔ1* and *sulI*) on the ORFs at the 3′-CS of a typical class 1 integron. The positive control for a typical class 1 integron used was *Acinetobacter baumannii* ATCC 19606. All primers and thermal cycling conditions are listed in Table 1.

### Mapping of integrons

In separate PCR assays, isolates positive for the *intI1* and *intI2* were assessed to detect their internal variable regions (IVRs). Specific primer set 5′-CS and 3′-CS, which joins the *attI1* site of the 5′-CS and the 3′-CS of *intI1,* was used fo*r intI1* positive isolates while the primer set hep74 and hep 51, which binds to the *attI2* and the *orfX* sites located downstream the GC regions, was used for *intI2* positive isolates, as indicated in Table 1. The PCR assays were carried out in triplicates to ensure reproducibility.

### Restriction analysis and DNA sequencing of amplicons

Amplicons of variable regions that appeared similar in size were exposed to the *AluI* restriction enzyme (Biolabs, England) for the restriction fragment length polymorphism (RFLP) to assess if the products were in the same sequence. For the identification of similar GC arrays in the integrons, *AluI* was chosen due to its recognition sequence being only four bases, thereby increasing the likelihood of its activity over the other enzymes that target the six-base sequence. Briefly, 30 µl of the amplicon was exposed to 1.0 µl of 10U/ml *AluI*. The reaction mixture was incubated for 4 h at 37 °C. The products were then run on a 2% agarose gel and visualized. These were characterized according to their distinct restriction profiles, and two randomly selected representative amplicons from each RFLP class were selected for sequencing. This step was taken to help reduce the risk of needlessly sequencing multiple identical variable regions.

### Variable sequence analysis, cassette identification

The content and arrangement of the inserted GCs within the amplified IVRs were analyzed through sequencing. The amplicons were sequenced in both directions on an ABI 3500XL sequencer using the Nimagen BrilliantDye™ Terminator Cycle sequencing kit V3.1 (Inqaba Biotechnical Industries, South Africa). The sequences were then modified with Chromas 2.7 and pairwise aligned using BioEdit sequence alignment editor software. BLAST nucleotide search analysis (https://blast.ncbi.nlm.nih.gov/Blast) was performed on the generated consensus sequences to identify the contents of the inserted gene cassette. The position of each gene in each cassette was determined using ABRicate 0.8.4 (https://github.com/tseemann/abricate) (ResFinder, ARG-ANNOT, CARD, and NCBI databases).

### Molecular typing of isolates

Enterobacterial Repetitive Intergenic Consensus PCR (ERIC-PCR) was done to evaluate the genotypic similarities of the integron-harbouring *K. pneumoniae* despite being isolated from different environmental matrices. PCR was performed using the primer sets ERIC1: (5′-ATGTAAGCTCCTGGGGATTCAC-3′) and ERIC 2: (5′ -AAGTAAGTGACTGGGG TGAGCG-3′) under conditions as described^46^. The amplicons were resolved in 3% agarose gel in a 0.5X TBE buffer and allowed to run at 100 V for 240 min.

### Statistical analysis

The descriptive statistical software in Microsoft Excel 2016 and the Statistical Package for the Social Sciences (SPSS) version 25 were used to examine the data (SPSS Inc., Chicago, IL). The data was validated, and relationships were calculated with 2 × 2 cross-tabulation tables utilizing exploratory data analysis. Pearson's Chi-square test was used to examine the statistical significance of susceptibility and the number of integron-positive isolates. A *P* value of less than 0.05 was considered significant.

Using Gelj v.2.0 software, computer-assisted pattern analysis was used to examine the DNA fingerprints generated from the ERIC-PCR. Pearson's correlation coefficient was used to compute the percentage similarity of digitized bands. The relatedness of the isolates was estimated using the unweighted pair group method with arithmetic mean (UPGMA) and complete linkage methods, which were shown as dendrograms.

## Results

### PCR confirmation of bacterial strains

From the 52 isolates that were positive for the *gryA* gene to confirm *Klebsiella* spp., 63% (33/52) were positive for *16S-23S ITS* to confirm *K. pneumoniae*. About 37% (19/52) of the remaining isolates were positive for the polygalacturonase *pehX* gene and identified as *K. oxytoca*.

### Prevalence of antibiotic resistance, ESBL and MBL production

**Figure 1:**
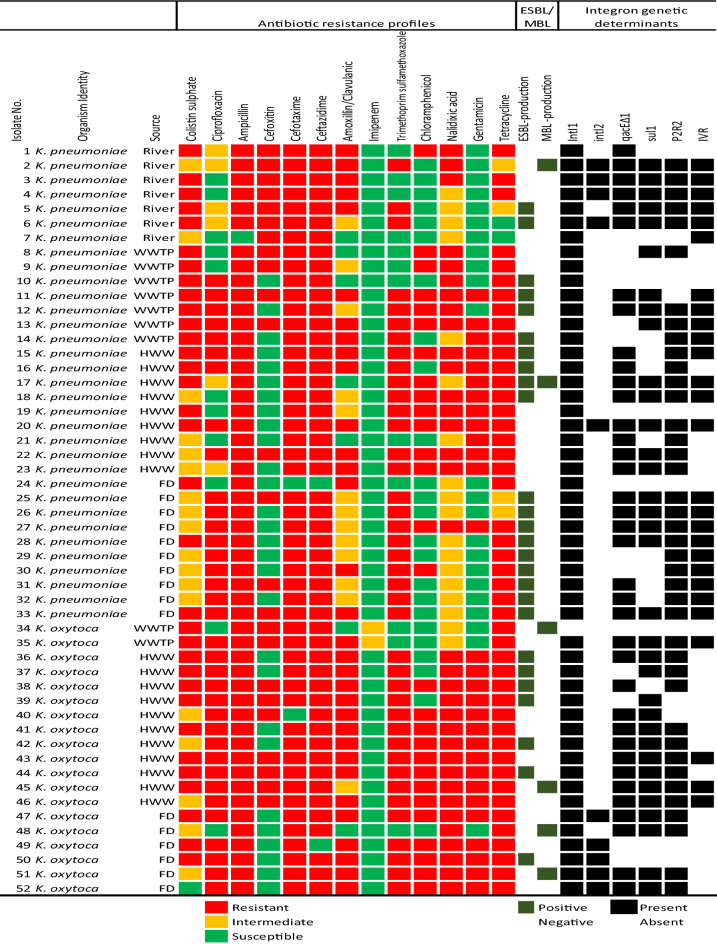
The resistance profiles, the production of ESBL and MBL, and the detection of integron genetic determinants of isolates from environmental matrices. Each isolate's susceptibility profiles to certain antibiotics are colour coded to indicate whether it is resistant, intermediate-resistant, or susceptible. The ESBL and MBL production is negative or positive, while the integron genetic elements are absent or present. *WWTP* Wastewater treatment plants effluent, *HWW* Hospital wastewater, *FD* Animal faecal droppings.

Figure 1 shows the antibiotic susceptibility fingerprints of each isolate to the 13 tested antibiotics. All the *K. pneumoniae* were resistant to nalidixic acid and colistin sulphate. It was followed keenly by their resistance to ampicillin, cefotaxime, and ceftazidime (98%). The percentage frequencies of resistance observed to the remaining antimicrobial agents assayed are as follows: tetracycline (94%), amoxicillin/clavulanic acid (85%), ciprofloxacin (73%), trimethoprim-sulfamethoxazole (73%), cefoxitin and chloramphenicol (45%), and gentamicin (39%). No resistance was observed to imipenem, a carbapenem, one of the drugs of last resort. All the *K. oxytoca* investigated exhibited resistance to nalidixic acid, ampicillin, and tetracycline. The least resistance (11%) was also observed to the imipenem. The remaining resistance frequencies were as follows: cefoxitin (47%), chloramphenicol (68%), trimethoprim-sulfamethoxazole and gentamicin (84%), ciprofloxacin, amoxicillin/clavulanic acid (89%), and colistin, cefotaxime, ceftazidime (95%). Here intermediate resistance observed was also classified as resistance. The percentage resistance frequencies of each species are shown in Fig. S1.

The DDST screening revealed that 50% (26/52) of the isolates were ESBL producers (Fig. 1). Of the 26 ESBL-producing isolates, 73% (19/26) were *K. pneumoniae*, while 27% (7/26) were *K. oxytoca*. For the MBL production, only 11.5% (6/52) of the isolates were positive, with two *K. pneumoniae* strains and four *K. oxytoca* strains. One *K. pneumoniae* strain isolated from hospital wastewater (Isolate No. 17) co-produced ESBL and MBL.

### Characterization of 5′ conserved segment (integrase + promoters)

Among the 52 confirmed *Klebsiella* spp., 98% (51/52) were integron-positive as they harboured the integron integrase (*intI1)* gene. All the *K. pneumoniae* harboured the *intI1* gene. The *intI1* gene was absent in only one *K. oxytoca* isolate, while 20% (10/51) of these *intI1* positive isolates concurrently harboured the *intI2* gene and were thus classified as class 1 + 2 integrons. Those carrying only the *intI1* gene classified as class 1 integrons were 80% (41/51). Notably, none of the isolates harboured the *intI2* gene only, as when present, they concurrently were found with *intI1*. No *intI3* gene for class 3 integrons was detected (Fig. 1). As shown in Table 2, detecting the integrase gene in each isolate was associated with resistance to three antibiotics, ciprofloxacin, amoxicillin-clavulanic acid, and imipenem at significant levels (P < 0.05).

**Table 2:** The association between resistance to antibiotics and the presence of integrons in *Klebsiella* spp.

Antibiotics	No. of resistant isolates		No. of susceptible isolates		*P*-value (χ^2^ test)
	Total (%)	Integron-positive (% of resistant isolates)	Total (%)	Integron-positive (% of susceptible isolates)	
Colistin sulphate	51 (98.1)	50 (98)	1 (2)	1 (2)	0.888
Ciprofloxacin	41 (78.8)	41 (80.4)	11 (21)	10 (20)	**0.051**
Ampicillin	51 (98.1)	50 (98)	1 (2)	1 (2)	0.888
Cefoxitin	24 (46.2)	23 (45.1)	28 (54)	28 (55)	0.275
Cefotaxime	50 (96.2)	49 (96.1)	2 (4)	2 (4)	0.840
Ceftazidime	50 (96.2)	49 (96.1)	2 (4)	2 (4)	0.840
Amoxicillin/clavulanic acid	45 (86.5)	45 (88.2)	7 (13)	6 (12)	**0.010**
Imipenem	2 (3.8)	1 (2)	50 (96)	50 (98)	**0.000**
Trimethoprim-sulfamethoxazole	40 (76.9)	40 (78.4)	12 (23)	11 (22)	0.065
Chloramphenicol	28 (53.8)	28 (54.9)	24 (46)	23 (45)	0.275
Nalidixic acid	52 (100)	52 (100)	0 (0)	0 (0)	^a^
Gentamicin	29 (55.8)	29 (56.9)	23 (44)	22 (43)	0.257
Tetracycline	50 (96.2)	49 (96.1)	2 (4)	2 (4)	0.840

Significant values are represented in bold.

^a^No statistics are computed because all the isolates displayed a 100% resistance to Nalidixic acid.

Of the 51 integrase-positive isolates examined, 78% (40/51) were positive for the *P2R2* investigated for a promoter gene on an integron located on the 5′ CS. Of these, 65% (26/40) were detected in *K. pneumoniae* isolates, while 35% (14/40) were harboured in *K. oxytoca* integrons. We could not amplify promoters targeting the *P2R2* on the remaining 11 integron-positive isolates.

### Characterization of the 3′ conserved segment (*qacEΔ1* + *sul1*)

For the ORFs located on 3′-CS, 80% (41/51) of the integron-positive isolates assayed harboured at least one of the *qacEΔ1* and *sul1*. Approximately 73% (37/51) were positive for the *qacEΔ1*, while 67% (34/51) had the *sulI*. All the *qacEΔ1* and *sul1-*positive isolates concurrently harboured both genes except four (Isolate No. 8, 13, 37 and 39). From the *intI1*-positive isolates, 20% (10/51) lacked any of the 3′-CS genes, while 14% (7/51) harboured the *qacEΔ1* without *sul1,* and 8% (4/51) were positive for *sul1* without *qacEΔ1*. About 65% (33/51) of the isolates were classified as typical class 1 integrons as they contained both genes on the 3′ CS.

### Characterization of internal variable regions (IVRs)

The internal variable regions that harbour GCs and are between the CS of the integron harbouring isolates were 53% (27/51). The mapping of *intI1* and *intI2* positive isolates revealed that 59% (22/37) and 50% (5/10) of the integrons were positive for IVRs, respectively. The *K. oxytoca* isolates with class 1 + 2 integrons did not harbour any IVRs. The amplified IVRs ranged from 160 to 1400 bp, with the mode being the least base pair. The analysis of the IVRs yielded eight amplicons of distinctly varied sizes as follows: ≈ 160 bp (n = 18), ≈ 190 bp (n = 2), ≈ 280 (n = 2), ≈ 350 (n = 1), ≈ 550 (n = 1), ≈ 700 bp (n = 1), ≈ 1200 (n = 1), and ≈ 1400 (n = 1).

**Figure 2:**
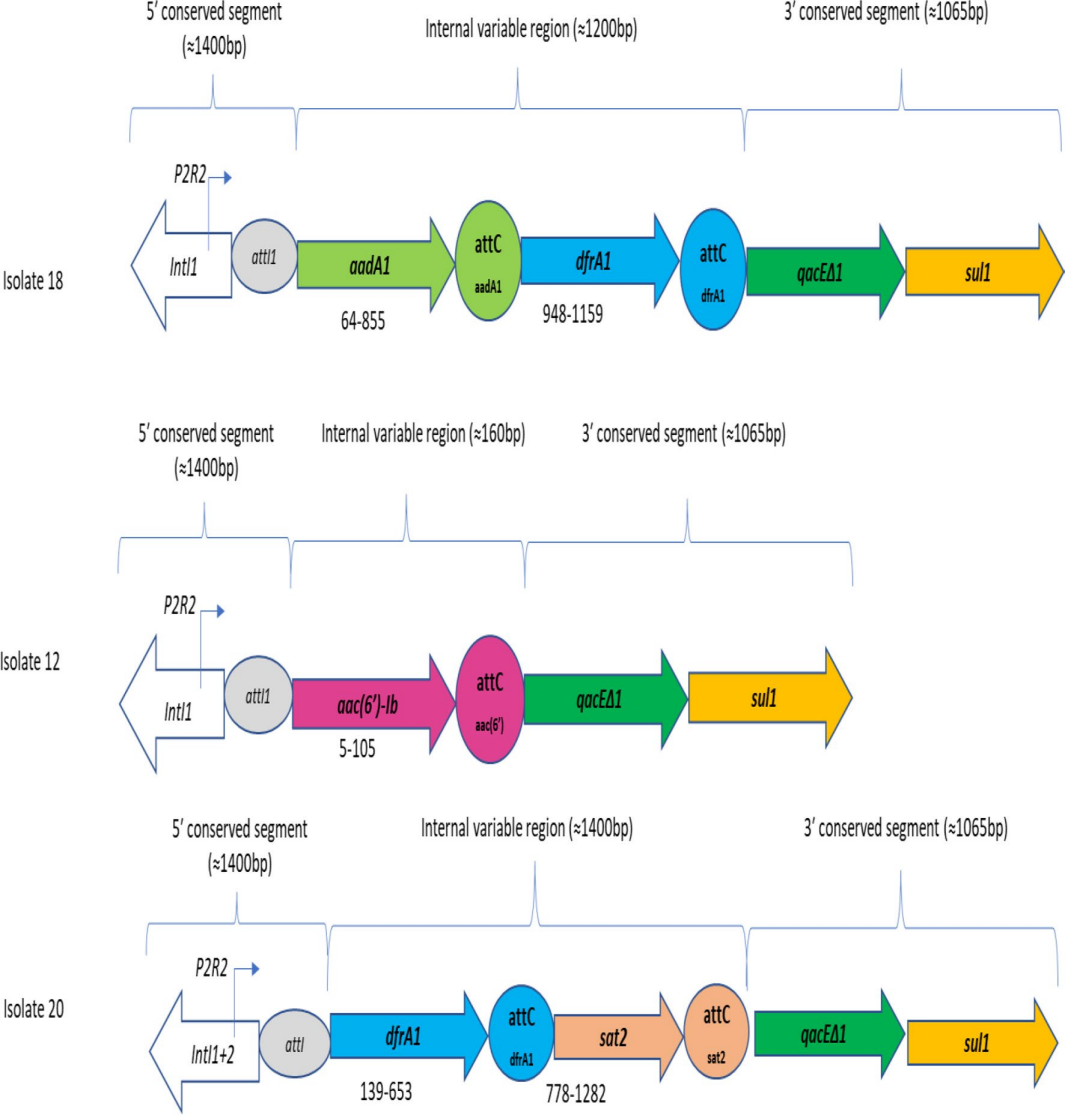
Schematic representation of some integron-positive *K. pneumoniae* with their associated gene cassette arrays. These isolates were positive for *intI1* and *intI1* + *2* for class 1 and class 1 + 2 integrons, respectively. The *P2R2* for the promoter is situated on the 5′ conserved segment. Also detected were *sul1* and *qacEΔ1* genes found on the 3′ conserved segment conferring resistance to sulphonamides and quaternary ammonium compounds, respectively. The cassette arrays include *the aadA1 and aac(6′)-Ib* gene, which encodes resistance to aminoglycosides. The *dfrA1* and *sat2* confer resistance to trimethoprim and streptothricin, respectively. The start and stop nucleotide sequences were also depicted, while the arrows indicate the transcription direction.

The analysis of the sequences yielded three distinct cassette arrays. These detected cassette arrays were *aac(6′)-Ib, aadA1*-*dfrA1*, and *dfrA1*-*sat2* (Table 3). They harboured genes that encode resistance to aminoglycosides, trimethoprim, and streptothricin. The most detected cassette was *aac(6′)-Ib* representing 82% (18/22) of all identified cassettes. The gene *dfrA1* was detected in 9% of the cassettes, while *sat2* and *aadA1* occurred only once. The sequences of the remaining IVR amplicons yielded empty or undetermined cassette arrays indicating they are likely to be novel integrons or integrons without any GCs, as they did not yield meaningful alignment when compared with the sequences in the GenBank. The schematic representation of some of the integrons and their associated gene cassette arrays are shown in Fig. 2.

**Table 3:** The content and arrangement of genes within the integrons' internal variable regions (n = 27).

Class of integron	No. of isolates	Cassette array	Approximate size (bp)	*qacEΔ1* + *sul1*
Class 1	16	*aac(6')-Ib*	160	+ 1^a^
	2	_^b^	190	+ 1
	1	*aadA1-dfrA1*	1200	+ 1
	1	_^b^	350	+ 1
	2	_^b^	280	+ 1^c^
Class 1 + 2	1	*dfrA1-sat2*	1400	+ 1
	2	aac(6')-Ib	160	+ 1
	1	_^b^	550	+ 1
	1	_^b^	700	+ 1

^a^Except for three isolates that were positive for *qacEΔ1* only, one isolate was positive for *sul1* only, while three of the isolates lacked both *qacEΔ1* + *sul1.*

^b^The amplicon yielded an undetermined/empty cassette array.

^c^One isolate lacked both *qacEΔ1* + *sul1.*

### Genetic relatedness of *K. pneumoniae* from various sources

**Figure 3:**
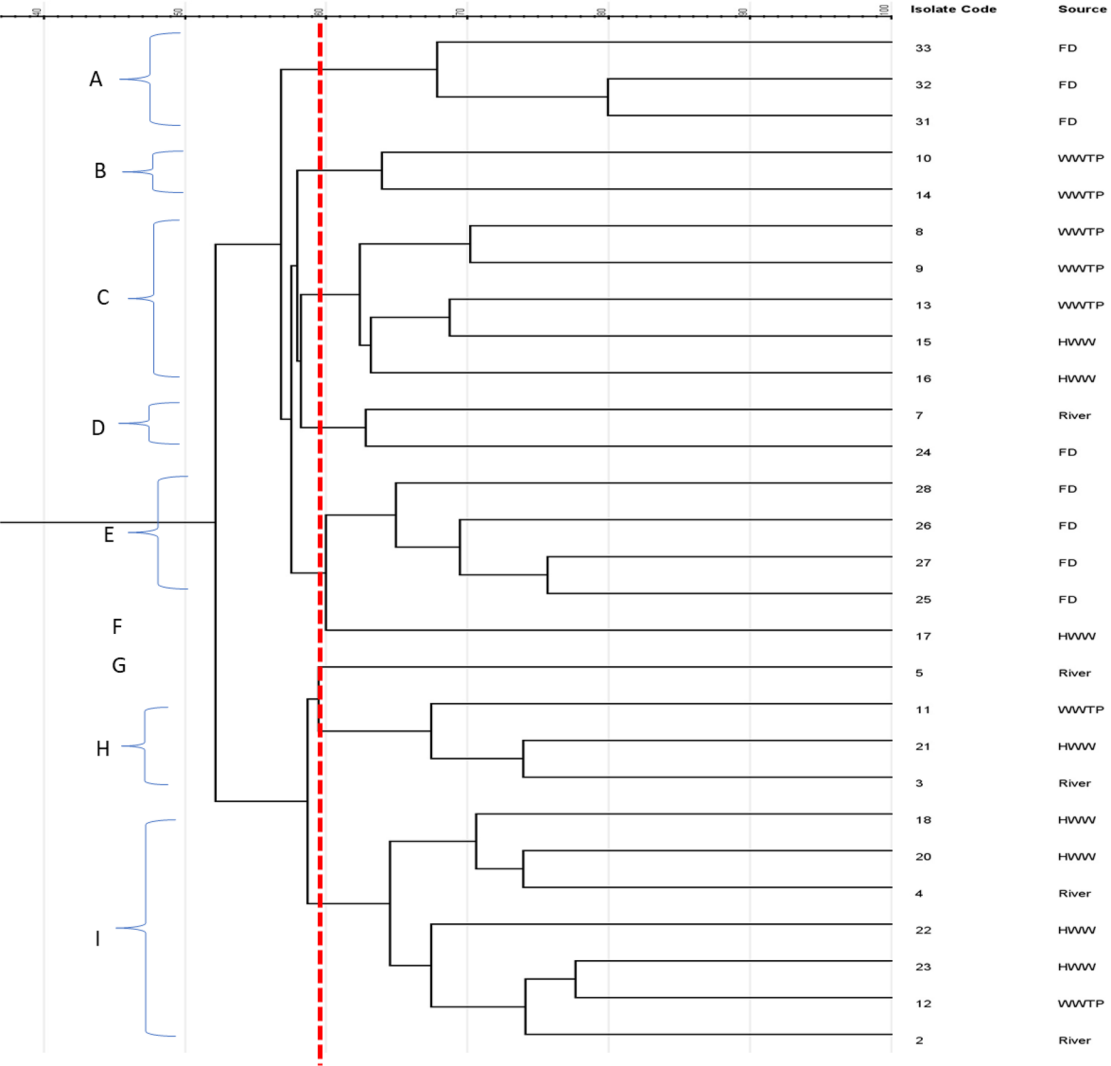
UPGMA dendrogram image obtained from clustering analysis indicating the genetic relatedness of *K. pneumoniae* (n = 28) recovered from various environmental sources using the ERIC-PCR technique. *HWW* Hospital wastewater, *WWTP* wastewater treatment plant, *FD* animal droppings.

The genotypic relatedness of all the *K. pneumoniae* investigated is indicated in the dendrogram generated from the ERIC-PCR fingerprinting of isolates. The fingerprints generated nine clusters (Fig. 3A–I) at a similarity cut-off value of 60%. Cluster I was the highest ERIC-genotype cluster. It comprises seven isolates with representatives from various water sources, with similarities ranging from 67 to 78%. Cluster C was grouped with five isolates from HWW and WWTP, while the least ERIC-genotype cluster was observed at F and G (one isolate from HWW and river, respectively). The eight FD isolates were spread into three clusters, three clustered at A while four clustered at E with 68% and 65% similarity indices. Cluster D was formed with an isolate each from the river and FD with 63% similarity. Cluster B contained two isolates from WWTP, while cluster H contained an isolate from WWTP, HWW, and river. It should be noted that 5 of the *K. pneumoniae* isolates did not generate any visible bands on the ERIC-PCR gel and were excluded from the analysis.

## Discussion

On speciation of the *Klebsiella* investigated in this study, *K. pneumoniae* (63%) was more frequently detected than *K. oxytoca* (37%). It agrees with the reports that *K. pneumoniae* is the most prevalent species of the genus as it is the causative agent of most nosocomial *Klebsiella* infections, thereby referred to as the most medically significant species of the genus^3, 47^. *K. pneumoniae* is becoming more recognized as an invasive and aggressive pathogen that carries some ARGs^48^. It has been reported to cause fatalities in various provinces within South Africa^49, 50^.

Antibiotic resistance was shown to be prevalent among the isolates in this investigation. They were all categorized as MDR because they were resistant to more than two distinct classes of antibiotics (Fig. 1). Many antibiotics are not entirely metabolized after consumption and end up in wastewater systems^34^. Antibiotics in wastewater have been demonstrated to impose selection pressure on antimicrobial-resistant bacteria, allowing them to spread to new environments^51^. This widespread usage of antimicrobials has resulted in an increased rate of antimicrobial resistance detection in bacteria. Almost all the isolates in this study were resistant to the β-lactams, including the third-generation cephalosporins. The least resistance was observed to imipenem, which is not usually a front-line prescription antibiotic. This low detection rate in resistance to carbapenems is somewhat expected and similar to reports of other studies where the resistance frequencies ranged from 7 to 15%^52, 53^. MDR *K. pneumoniae* has emerged as a critical problem in treating nosocomial infections worldwide^2^; therefore, its environmental detection is quite concerning. The production of β-lactamase by *Klebsiella* spp. is one of their resistance mechanisms to β-lactam antibiotics^54^. Our study revealed a prevalence phenotype of 50% and 12% of ESBL and MBL, respectively. These β-lactamases are also associated with co-resistance to other antibiotics by bacterial species harbouring them, further limiting possible treatment options. These β-lactamases could be carried on mobile genetic elements such as integrons, which aid in the horizontal spread of ARGs among diverse bacterial species^48, 54, 55^. In a study in China, clinically relevant strains of ESBL-producing *K. pneumoniae* were also found in diverse environmental sources^7^. They also reported that all the isolates were MDR, with a few being clinically relevant strains known to cause hospital outbreaks. These studies, including ours, aim to draw our attention to the need to ascertain the involvement of the environment in the spread of these pathogens and the concomitant risk to public health.

In this study, integrons were detected in all but one of the isolates investigated, with class 1 integrons being most predominant. The studies of^2^ and^18^ reported that all their MDR *K. pneumoniae* harboured the *intI* gene, although these isolates were from clinical samples. Other research from environmental origins has also reported the detection of the *intI1* gene as the most prevalent in similar studies^13, 22, 56^. Furthermore, the predominance of *intI1* in this study is similar to other studies reporting that class 1 integrons are more prevalent than class 2 integrons in Gram-negative bacteria^31, 56–59^. There is a strong association between the occurrence of integrons and the prevalence of MDR in Gram-negative bacteria due to their high capacity for transferring antimicrobial resistance genes^31, 56, 60^. Although our results indicate a significant association between *intI1* and three out of thirteen antibiotics investigated (Table 3), in research by Li and colleagues^61^, integron harbouring isolates demonstrated resistance to a substantially greater number of antibiotics than negative isolates. Other investigations have found a high prevalence of integron-positive MDR *Klebsiella* spp.^2, 62^. Integrons give a selective advantage to bacteria in settings where antibiotic use causes selective pressures, which may explain the high occurrence of integrons among MDR strains.

Typical class 1 integrons usually harbour quaternary ammonium compounds and sulphonamide resistance genes^20, 63^. In this study, 59% (30/51) of the integrase-positive isolates harboured both *sul1* and *qacEΔ1*, further confirming the presence of the 3′ CS of a typical class 1 integron which was also reported in similar studies^56, 57^. It was observed that 27% (8/30) of these isolates concurrently have the *intI2* gene (Fig. 1). Invariably, this shows that it is possible that some bacteria with a typical class 1 integron can still harbour the class 2 integrase gene, a scenario that has been previously reported^56^. Furthermore, we report a prevalence of 78% of a promoter type, the *P2R2*, located on the integron. It further confirms that an integron can act as an expression vector as this promoter can be involved in the expression of the genes in the cassette arrays acquired from the environment once incorporated into the integrons. The inability to detect the *P2R2* on the remaining isolates via PCR could have been due to a mutation in the promoter gene located within the integrase, or those integrons could have a promoter type different from the assayed gene.

Three unique GC arrays in the integrons are shown in Table 2. These results prove that environmental sources also serve as a potential repertoire of mobile genetic elements harbouring different antibiotic resistance GCs. The GCs contained genes encoding resistance to trimethoprim (*dfrA1*), streptothricin (*sat2*), and aminoglycosides (*aadA1* and *aac(6)-Ib*). The most prevalent GC was the *aac(6)-Ib,* which occurred singly. Our results indicate that 53% (27/51) of the isolates harbouring the *intI1* contained the internal variable regions (Fig. 1). The *aac(6)-Ib* encodes an enzyme 6′-N-acetyltransferase and belongs to the family of aminoglycoside acetyltransferases, while the *aadA1* encodes the aminoglycoside adenyl transferase. They confer resistance to aminoglycosides such as amikacin, gentamicin, tobramycin, neomycin, and streptomycin. The *Sat2* encodes streptothricin-N-acetyltransferase, which confers resistance to streptothricin. The detection of the *dfrA* encodes dihydrofolate reductase, which confers resistance to trimethoprim, has been associated with selection pressure and high use of trimethoprim, especially in clinical sources^2^. However, this study infers that such a selective burden may equally exist in contaminated aquatic environments.

According to the sequencing results, 13.7% of these isolates did not contain any GCs (Table 3), and these could be a result of defects or mutations at the 3′ CS or the GCs belonging to an unusual or complex class 1 integrons^31, 64^. The detection of integrons with empty GCs has been reported, and it shows the readiness of such isolates to capture GCs readily and subsequently express the ARGs harboured on them. The ERIC-PCR dendrogram image, as seen in Fig. 3, shows the clonal relatedness of the integron-positive *K. pneumoniae* from the various sources clustered in similar groups, indicating high genetic relatedness among the integron-bearing MDR isolates. This further reiterates the ability of bacterial species from various niches to exchange genetic elements, which can confer specific adaptability properties.

## Conclusions

MDR bacterial strains have been widely disseminated due to the distribution of antibiotic-resistant strains, particularly the ESBL and carbapenemase producers. The occurrence of integrons in these MDR bacterial strains has further accelerated the spread of ARGs into diverse environmental sources. The data obtained from this study show that integrons with their associated gene cassettes are widely distributed in *Klebsiella* spp. from environmental matrices that may further constitute a problem when treating bacterial infections. The baseline properties of integrons in *Klebsiella* species isolated from several environmental matrices in the Eastern Cape Province, South Africa, have never been previously reported.

### Supplementary Information

Supplementary Figure S1.

## Data Availability

The original contributions presented in the study are included in the article/Supplementary Material. Further enquiries can be directed to the corresponding author.
