# Supplementary material for: Prevalence, molecular characterization of integrons and its associated gene cassettes in Klebsiella pneumoniae and K. oxytoca recovered from diverse environmental matrices
Source: Sci Rep. 2023 Sep 1;13:14373. doi: 10.1038/s41598-023-41591-7 (PMC10474106; doi:10.1038/s41598-023-41591-7)
Supplement: Supplementary file 1 — Supplementary Figure S1. [file 41598_2023_41591_MOESM1_ESM.docx]

**
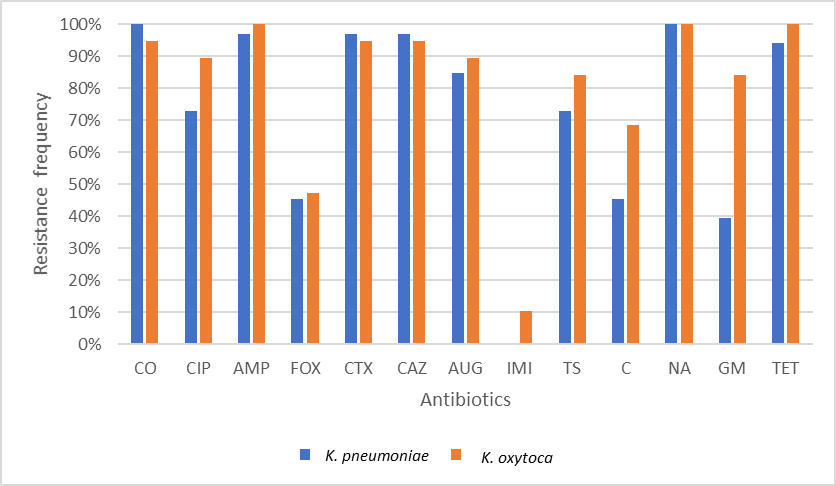
**

**S1 Fig . Total resistance frequencies of *K. pneumoniae* (n=33) and *K. oxytoca* (n=19) against 13 antibiotics**. Antibiotics code: CTX-cefotaxime, CO-colistin sulphate, CIP-ciprofloxacin, TS-trimethoprim/sulfamethoxazole, FOX-cefoxitin, NA-nalidixic acid, AMP-ampicillin, AUG-amoxicillin/clavulanic acid, IMI-imipenem, C-chloramphenicol, GM-gentamicin, CAZ-ceftazidime, and TET-tetracycline.
